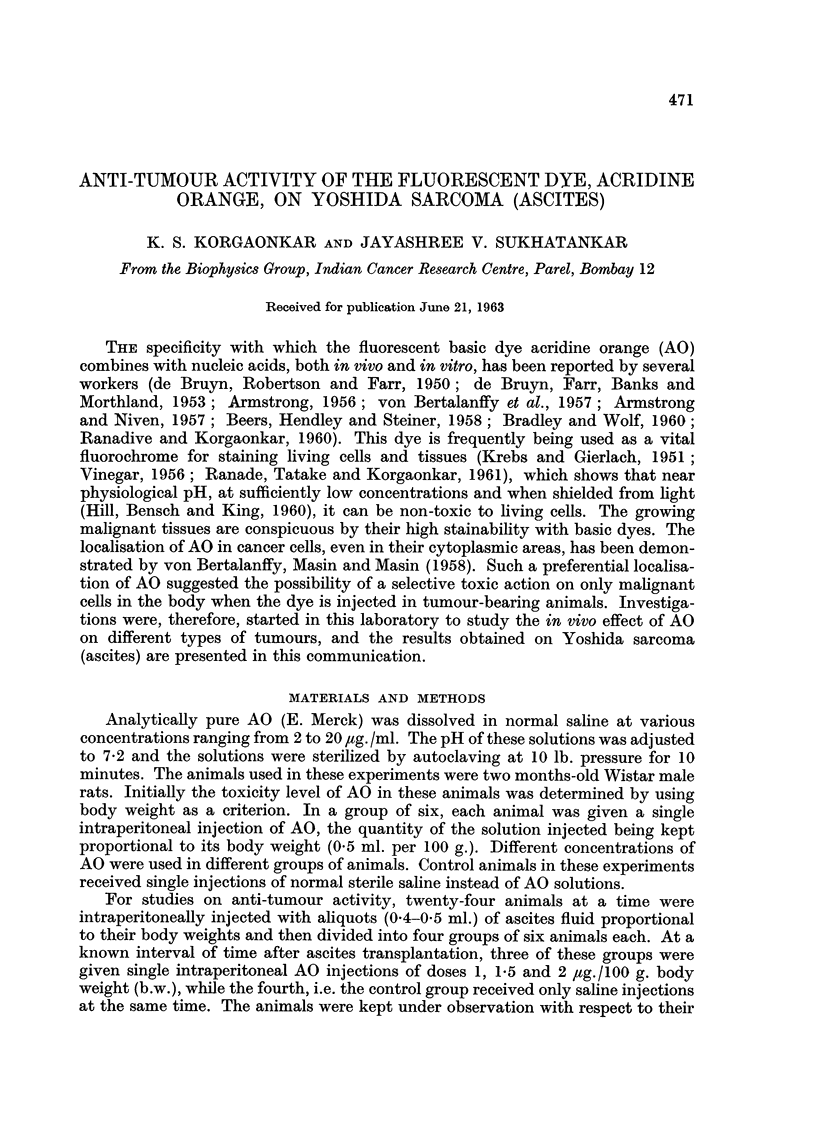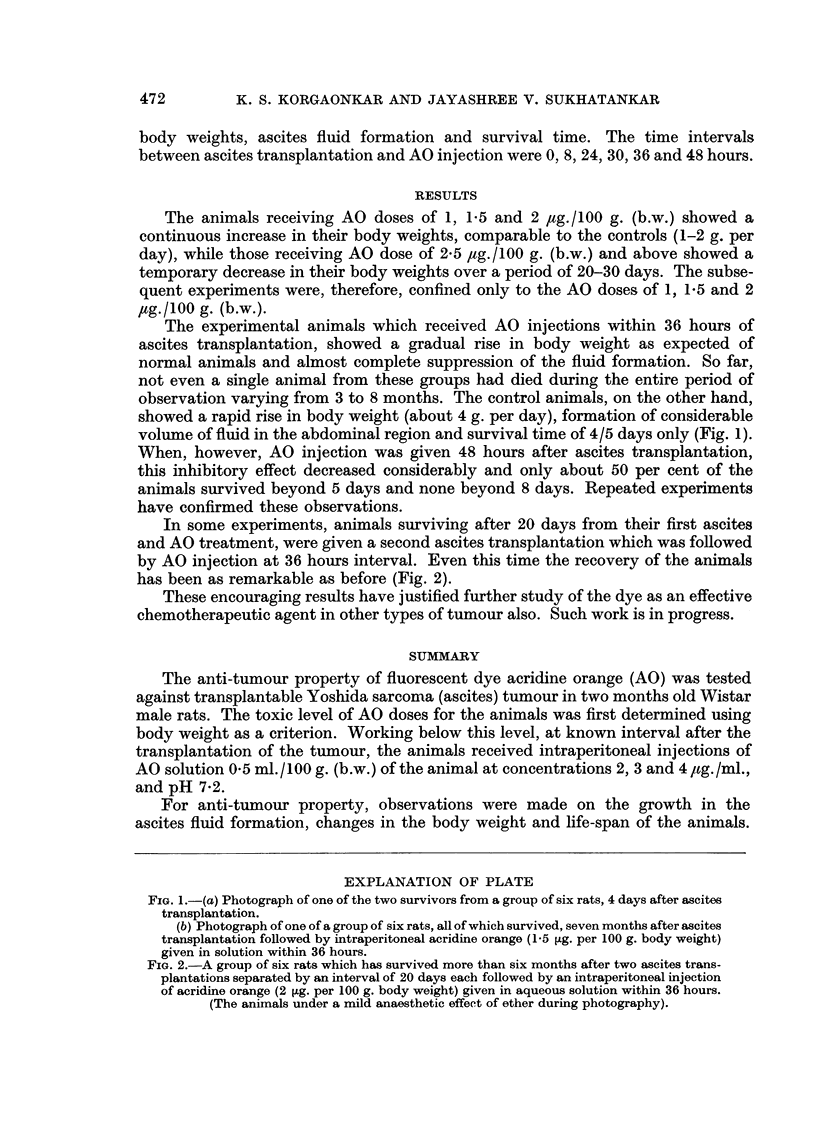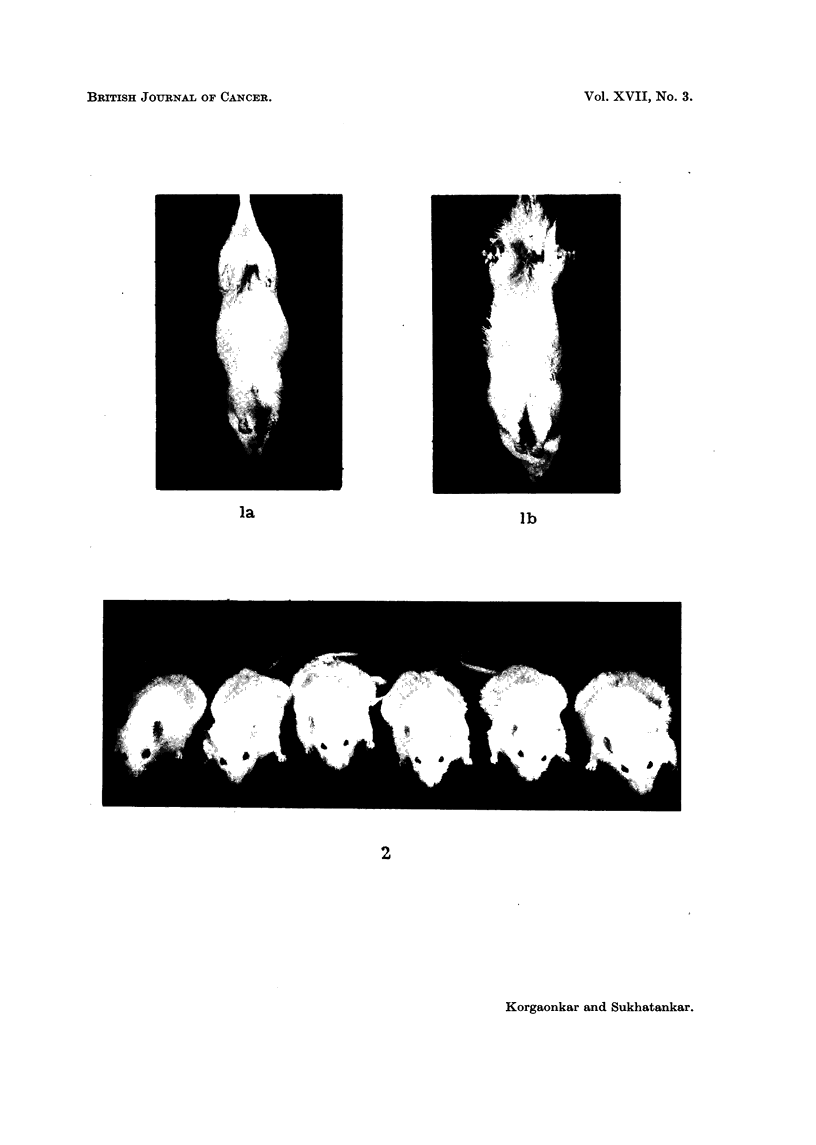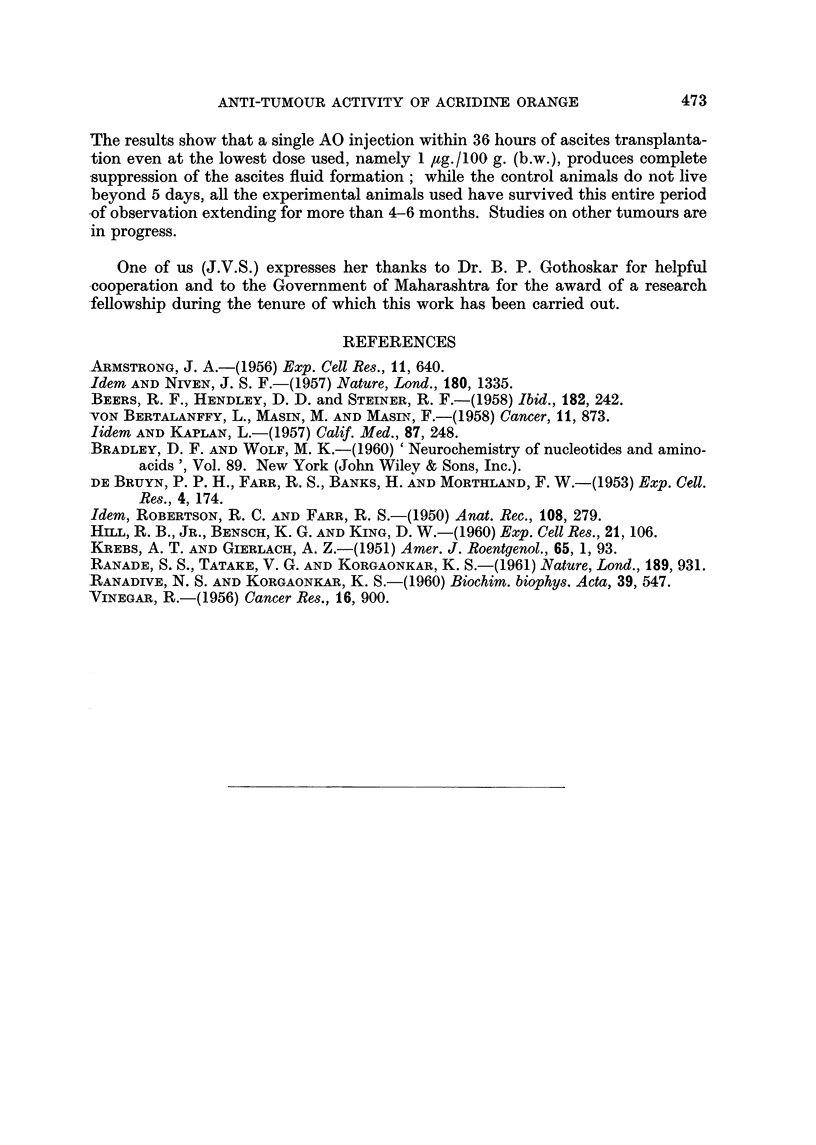# Anti-Tumour Activity of the Fluorescent Dye, Acridine Orange, on Yoshida Sarcoma (Ascites)

**DOI:** 10.1038/bjc.1963.62

**Published:** 1963-09

**Authors:** K. S. Korgaonkar, Jayashree V. Sukhatankar

## Abstract

**Images:**


### ANTI-TUMOUR ACTIVITY OF THE FLUORESCENT DYE, ACRIDINE ORANGE, ON YOSHIDA SARCOMA (ASCITES)

K. S. KORGAONKAR AND JAYASHREE V. SUKHATANKAR

*From the Biophysics Group, Indian Cancer Research Centre, Parel, Bombay* 12

Received for publication June 21, 1963

THE specificity with which the fluorescent basic dye acridine orange (AO) combines with nucleic acids, both *in vivo* and *in vitro*, has been reported by several workers (de Bruyn, Robertson and Farr, 1950; de Bruyn, Farr, Banks and Morthland, 1953; Armstrong, 1956; von Bertalanffy *et al.*, 1957; Armstrong and Niven, 1957; Beers, Hendley and Steiner, 1958; Bradley and Wolf, 1960; Ranadive and Korgaonkar, 1960). This dye is frequently being used as a vital fluorochrome for staining living cells and tissues (Krebs and Gierlach, 1951; Vinegar, 1956; Ranade, Tatake and Korgaonkar, 1961), which shows that near physiological pH, at sufficiently low concentrations and when shielded from light (Hill, Bensch and King, 1960), it can be non-toxic to living cells. The growing malignant tissues are conspicuous by their high stainability with basic dyes. The localisation of AO in cancer cells, even in their cytoplasmic areas, has been demonstrated by von Bertalanffy, Masin and Masin (1958). Such a preferential localisation of AO suggested the possibility of a selective toxic action on only malignant cells in the body when the dye is injected in tumour-bearing animals. Investigations were, therefore, started in this laboratory to study the *in vivo* effect of AO on different types of tumours, and the results obtained on Yoshida sarcoma (ascites) are presented in this communication.

### MATERIALS AND METHODS

Analytically pure AO (E. Merck) was dissolved in normal saline at various concentrations ranging from 2 to 20 $\mu$g./ml. The pH of these solutions was adjusted to 7·2 and the solutions were sterilized by autoclaving at 10 lb. pressure for 10 minutes. The animals used in these experiments were two months-old Wistar male rats. Initially the toxicity level of AO in these animals was determined by using body weight as a criterion. In a group of six, each animal was given a single intraperitoneal injection of AO, the quantity of the solution injected being kept proportional to its body weight (0·5 ml. per 100 g.). Different concentrations of AO were used in different groups of animals. Control animals in these experiments received single injections of normal sterile saline instead of AO solutions.

For studies on anti-tumour activity, twenty-four animals at a time were intraperitoneally injected with aliquots (0·4–0·5 ml.) of ascites fluid proportional to their body weights and then divided into four groups of six animals each. At a known interval of time after ascites transplantation, three of these groups were given single intraperitoneal AO injections of doses 1, 1·5 and 2 $\mu$g./100 g. body weight (b.w.), while the fourth, i.e. the control group received only saline injections at the same time. The animals were kept under observation with respect to their body weights, ascites fluid formation and survival time. The time intervals between ascites transplantation and AO injection were 0, 8, 24, 30, 36 and 48 hours.

### RESULTS

The animals receiving AO doses of 1, 1·5 and 2 $\mu$g./100 g. (b.w.) showed a continuous increase in their body weights, comparable to the controls (1–2 g. per day), while those receiving AO dose of 2·5 $\mu$g./100 g. (b.w.) and above showed a temporary decrease in their body weights over a period of 20–30 days. The subsequent experiments were, therefore, confined only to the AO doses of 1, 1·5 and 2 $\mu$g./100 g. (b.w.).

The experimental animals which received AO injections within 36 hours of ascites transplantation, showed a gradual rise in body weight as expected of normal animals and almost complete suppression of the fluid formation. So far, not even a single animal from these groups had died during the entire period of observation varying from 3 to 8 months. The control animals, on the other hand, showed a rapid rise in body weight (about 4 g. per day), formation of considerable volume of fluid in the abdominal region and survival time of 4/5 days only (Fig. 1). When, however, AO injection was given 48 hours after ascites transplantation, this inhibitory effect decreased considerably and only about 50 per cent of the animals survived beyond 5 days and none beyond 8 days. Repeated experiments have confirmed these observations.

In some experiments, animals surviving after 20 days from their first ascites and AO treatment, were given a second ascites transplantation which was followed by AO injection at 36 hours interval. Even this time the recovery of the animals has been as remarkable as before (Fig. 2).

These encouraging results have justified further study of the dye as an effective chemotherapeutic agent in other types of tumour also. Such work is in progress.

### SUMMARY

The anti-tumour property of fluorescent dye acridine orange (AO) was tested against transplantable Yoshida sarcoma (ascites) tumour in two months old Wistar male rats. The toxic level of AO doses for the animals was first determined using body weight as a criterion. Working below this level, at known interval after the transplantation of the tumour, the animals received intraperitoneal injections of AO solution 0·5 ml./100 g. (b.w.) of the animal at concentrations 2, 3 and 4 $\mu$g./ml., and pH 7·2.

For anti-tumour property, observations were made on the growth in the ascites fluid formation, changes in the body weight and life-span of the animals.

---

### EXPLANATION OF PLATE

FIG. 1.—(*a*) Photograph of one of the two survivors from a group of six rats, 4 days after ascites transplantation.

(*b*) Photograph of one of a group of six rats, all of which survived, seven months after ascites transplantation followed by intraperitoneal acridine orange (1·5 $\mu$g. per 100 g. body weight) given in solution within 36 hours.

FIG. 2.—A group of six rats which has survived more than six months after two ascites transplantations separated by an interval of 20 days each followed by an intraperitoneal injection of acridine orange (2 $\mu$g. per 100 g. body weight) given in aqueous solution within 36 hours. (The animals under a mild anaesthetic effect of ether during photography).

1a

1b

2

Korgaonkar and Sukhatankar.

The results show that a single AO injection within 36 hours of ascites transplantation even at the lowest dose used, namely 1 $\mu$g./100 g. (b.w.), produces complete suppression of the ascites fluid formation ; while the control animals do not live beyond 5 days, all the experimental animals used have survived this entire period of observation extending for more than 4–6 months. Studies on other tumours are in progress.

One of us (J.V.S.) expresses her thanks to Dr. B. P. Gothoskar for helpful cooperation and to the Government of Maharashtra for the award of a research fellowship during the tenure of which this work has been carried out.

### REFERENCES

Armstrong, J. A.—(1956) *Exp. Cell Res.*, **11**, 640.
*Idem* and Niven, J. S. F.—(1957) *Nature, Lond.*, **180**, 1335.
Beers, R. F., Hendley, D. D. and Steiner, R. F.—(1958) *Ibid.*, **182**, 242.
von Bertalanffy, L., Masin, M. and Masin, F.—(1958) *Cancer*, **11**, 873.
*Iidem* and Kaplan, L.—(1957) *Calif. Med.*, **87**, 248.
Bradley, D. F. and Wolf, M. K.—(1960) ' Neurochemistry of nucleotides and amino-acids ', Vol. 89. New York (John Wiley & Sons, Inc.).
de Bruyn, P. P. H., Farr, R. S., Banks, H. and Morthland, F. W.—(1953) *Exp. Cell. Res.*, **4**, 174.
*Idem*, Robertson, R. C. and Farr, R. S.—(1950) *Anat. Rec.*, **108**, 279.
Hill, R. B., Jr., Bensch, K. G. and King, D. W.—(1960) *Exp. Cell Res.*, **21**, 106.
Krebs, A. T. and Gierlach, A. Z.—(1951) *Amer. J. Roentgenol.*, **65**, 1, 93.
Ranade, S. S., Tatake, V. G. and Korgaonkar, K. S.—(1961) *Nature, Lond.*, **189**, 931.
Ranadive, N. S. and Korgaonkar, K. S.—(1960) *Biochim. biophys. Acta*, **39**, 547.
Vinegar, R.—(1956) *Cancer Res.*, **16**, 900.